# The prion-like RNA-processing protein HNRPDL forms inherently toxic amyloid-like inclusion bodies in bacteria

**DOI:** 10.1186/s12934-015-0284-7

**Published:** 2015-07-11

**Authors:** Susanna Navarro, Patrizia Marinelli, Marta Diaz-Caballero, Salvador Ventura

**Affiliations:** Institut de Biotecnologia i Biomedicina, Departament de Bioquimica i Biologia Molecular, Universitat Autònoma de Barcelona, Bellaterra, 08193 Barcelona, Spain

**Keywords:** Inclusion bodies, Bacteria, Amyloid, Prions, Prion-like domains, Heterogeneous ribonucleoproteins, Neurodegenerative disorders

## Abstract

**Background:**

The formation of protein inclusions is connected to the onset of many human diseases. Human RNA binding proteins containing intrinsically disordered regions with an amino acid composition resembling those of yeast prion domains, like TDP-43 or FUS, are being found to aggregate in different neurodegenerative disorders. The structure of the intracellular inclusions formed by these proteins is still unclear and whether these deposits have an amyloid nature or not is a matter of debate. Recently, the aggregation of TDP-43 has been modelled in bacteria, showing that TDP-43 inclusion bodies (IBs) are amorphous but intrinsically neurotoxic. This observation raises the question of whether it is indeed the lack of an ordered structure in these human prion-like protein aggregates the underlying cause of their toxicity in different pathological states.

**Results:**

Here we characterize the IBs formed by the human prion-like RNA-processing protein HNRPDL. HNRPDL is linked to the development of limb-girdle muscular dystrophy 1G and shares domain architecture with TDP-43. We show that HNRPDL IBs display characteristic amyloid hallmarks, since these aggregates bind to amyloid dyes in vitro and inside the cell, they are enriched in intermolecular β-sheet conformation and contain inner amyloid-like fibrillar structure. In addition, despite their ordered structure, HNRPDL IBs are highly neurotoxic.

**Conclusions:**

Our results suggest that at least some of the disorders caused by the aggregation of human prion-like proteins would rely on the formation of classical amyloid assemblies rather than being caused by amorphous aggregates. They also illustrate the power of microbial cell factories to model amyloid aggregation.

**Electronic supplementary material:**

The online version of this article (doi:10.1186/s12934-015-0284-7) contains supplementary material, which is available to authorized users.

## Background

Protein misfolding and aggregation into amyloid conformations is linked to the onset of a growing number of human disorders, from neurodegenerative diseases such as Alzheimer’s, through transmissible prionic encephalopathies, to non-neurodegenerative amyloidoses such as type II diabetes [1–3]. The proteins involved in the onset of these disorders are not related in terms of sequence and/or structure and, in fact, the population of amyloid compatible conformations seems to be a generic property of many polypeptides [4]. Accordingly, the ability to sequester potentially harmful misfolded proteins into insoluble intracellular deposits appears to be a mechanism conserved throughout the evolution, from prokaryotic to higher organisms [5–9]. In bacteria, misfolded polypeptides are accumulated into inclusion bodies (IBs), insoluble aggregates usually located at the cell poles [10, 11]. The formation of IBs in bacteria has long been regarded as an unspecific process depending on the establishment of hydrophobic contacts between partially or totally unfolded species after protein synthesis at the ribosome [12]. However, an increasing body of evidence indicates that bacterial IBs share a number of common structural features with the highly ordered and, in many cases, pathogenic amyloid fibrils, specially when amyloidogenic proteins are recombinantly expressed [13, 14], as for the cases of Aβ peptide [15, 16] and Tau [17] proteins linked to Alzheimer’s disease or the polyglutamine-containing Ataxin-3 protein associated with the Machado-Joseph disease [18]. Therefore, IBs have become an attractive model to study intracellular protein aggregation and their consequences in simple but biologically relevant conditions that cannot be easily recapitulated in vitro, such as continuous synthesis of the amyloidogenic protein of interest, the presence of the quality control machinery or a naturally highly crowded environment [19–21].

Prion proteins are a particularly intriguing type of amyloids, since their aggregated states have a self-perpetuating ability. Het-s, from the fungus *Podospora anserina*, was the first prion protein whose bacterial IBs were shown to display amyloid-like properties [22, 23]. When bacterial Het-s IBs were transfected into prion-free fungal strains, they promoted prionic conversion at levels comparable to those induced by homologous in vitro formed amyloid fibrils [22]. In yeast, several polypeptides can form prions that behave as dominant non-Mendelian cytoplasmic genetic elements [24–26]. The best-characterized yeast prionogenic proteins are Sup35 and Ure2p, which, in their aggregated state, form two cytosolic inheritable elements named PSI+ and URE3, respectively. We have exploited microbial cell factories to show that these two proteins form amyloid-like IBs when they are recombinantly expressed [27]. As in the case of Het-s, purified bacterial Sup35 IBs induce the acquisition of the prion phenotype when transfected in prion-free yeast strains [27–29]. These observations confirm that the IBs molecular structure highly resembles to the fine architecture of fibrils, in such a way that even the propagating properties of amyloids, which depend on a very specific conformational signature, appear to be shared by the two types of aggregates.

A common feature of most described yeast prions is the presence of a distinctive prion domain (PrD) [26]. Typically, these domains display sequences of low complexity, highly enriched in asparagine (N) and/or glutamine (Q) residues and are predicted to be intrinsically unstructured [30]. Yeast PrDs can switch between this unfolded conformation and a transmissible cross-β conformation, being both necessary and sufficient for amyloid formation and propagation [31]. Interestingly, protein domains enriched in Q/N residues are over-represented in eukaryotic genomes, including the human genome, relative to prokaryotic ones, suggesting that prion-like conformational conversion might have evolved as a mechanism for regulating functionality in eukaryotic proteins [32]. Around 250 human proteins have been identified with regions similar to the yeast PrDs regarding to amino acid composition [33–36]. Several of these proteins containing Prion Like Domains (PrLDs) have recently been linked to different neurodegenerative disorders in humans, suggesting that they are potentially pathogenic [37, 38]. Most of them are RNA-binding proteins that form inclusions in affected patients. So far, they include: (1) fused in sarcoma (FUS), TAR DNA-binding protein 43 (TDP-43), EWSR1 and TAF15, involved in amyotrophic lateral sclerosis (ALS) and/or some forms of frontotemporal lobar degeneration (FTLD) [39–42], (2) hnRNPA2B1 and hnRNPA1, linked to familial inclusion body myopathy with Paget’s disease of bone, frontotemporal dementia and ALS [43] and (3) TIA1, a protein associated with Welander distal myopathy [44].

Despite TDP-43 is perhaps the best characterized of these PrLDs-containing proteins it still not clear whether the pathological aggregates formed by this protein have an amorphous or an amyloid nature. The difficulty of purifying soluble TDP-43 makes challenging to decipher this issue by means of classical in vitro aggregation studies. Chiti and co-workers have circumvented this limitation using bacteria to model intracellular TDP-43 aggregation. Interestingly, despite TDP-43 IBs were toxic to neuroblastoma, they didn’t exhibit amyloid signatures and were structurally amorphous [45]. To address whether this lack of ordered structure in protein deposits is a common property of pathogenic human PrLD-containing proteins we characterize here the IBs formed by heterogeneous nuclear ribonucleoprotein D-like (HNRPDL), a heterogeneous ribonucleoprotein (hnRNP) family member [46]. HNRPDL is predicted to contain a PrLD at its C-terminus and it has been recently shown to be linked to limb-girdle muscular dystrophy 1G, a genetically determined muscle disorder with a primary or predominant involvement of the pelvic or shoulder girdle musculature [47]. We show here that whereas, as in the case of TDP-43, HNRPDL IBs are inherently toxic to neuroblastoma cells, they display clear amyloid features, suggesting that at least some of the disorders caused by these human prion-like proteins might rely on the formation of structured amyloid assemblies.

## Results

### HNRPDL displays a predicted amyloidogenic prion-like domain at the C-terminus

**Figure 1:**
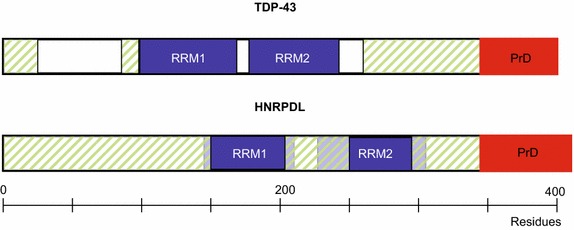
TDP-43 and HNRPDL domain architecture. Cartoons of proteins TDP-43 and HNRPDL show the domain architecture, where RRM accounts for RNA recognition motif and are represented in *blue*, and predicted disordered regions and prion domains (PrD) are shown in* striped green* and *red*, respectively. The places where RRM domains as assigned according to PFAM overlay with disordered predicted regions were assumed to correspond to canonical RRM domains.

The heterogeneous nuclear ribonucleoprotein d-like, also known as HNRPDL, belongs to the subfamily of ubiquitously expressed heterogeneous nuclear ribonucleoproteins (hnRNPs). These proteins are associated with pre-mRNAs in the nucleus, functioning in mRNA biogenesis and mRNA metabolism [46]. Although all of the hnRNPs are present in the nucleus, some shuttle between the nucleus and the cytoplasm [48]. HNRPDL is a 420 residues long protein for which no structural information is available yet. Both SMART (http://smart.embl-heidelberg.de) and PFAM (pfam.sanger.ac.uk/) databases coincide to indicate the presence of two contiguous canonical RNA recognition motifs (RRM) including residues 149–221 and 234–306, occupying a central position in the protein (Figure 1). Both the N- and C- terminal boundaries of these small domains are predicted to be low complexity regions without any associated function or structural motif. Disorder predictions using FoldIndex [49], FoldUnfold [50], and RONN [51] algorithms suggest that both the 1–149 and 306–420 sequence stretches are essentially disordered (Figure 1). The amino acid compositional bias of Q/N enriched prion domains has allowed the recent development of three different algorithms to identify the presence of PrLDs in protein sequences: PAPA [52], PLAAC [53] and PrionScan [54]. No prionic propensity is predicted with any of these programs for the N-terminal segment, whereas all of them identify the C-terminal region as displaying a PrLD comprising residues 340–420. Overall, this domain architecture and PrLD location recapitulates that of TDP-43 (Figure 1; Table 1).

**Table 1:** Prediction of PRLDs and their amyloid cores potency in the sequences of HNRPDL and TDP-43 RNA-binding proteins

	Uniprot	PrD			pWaltz score [34]
		PAPA [52]	PLAAC [53]	Prion Scan [54]	
HNRPDL	O14979	337–417	339–418	341–420	82.27
TDP-43	Q13148	346–416	299–378	341–417	68.16

We have recently shown that the identification and evaluation of the potency of amyloid nucleating sequences in the context of disordered Q/N rich protein segments allows discrimination of genuine yeast prions from non-prionic sequences displaying very similar amino acid composition, a concept that was implemented in the pWALTZ algorithm [34]. The C-terminal PrLD of HNRPDL displays a pWALTZ score (82.27) higher than the corresponding PrLD in TDP-43 (68.16) (Table 1) and, strikingly, higher than those of Ure2p (73.99) and Sup35 (73.66) prion domains [34], thus indicating the presence of an amyloidogenic sequence stretch comprising residues 342–362 in this Q/N rich disordered protein region.

### Aggregation of HNRPDL into IBs in bacteria

**Figure 2:**
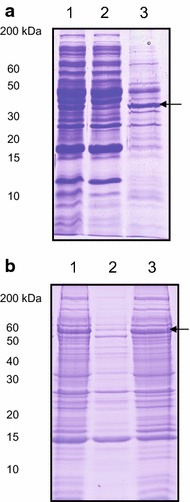
Expression of recombinant HNRPDL protein in *E. coli* cells. **a** Analysis on SDS-PAGE of *E. coli* cells extracts expressing HNRPDL protein. **b** SDS-PAGE analysis of cell extracts from cells expressing the GST-HNRPDL fusion. On both gels *lane 1* shows total extract; *lane 2*, soluble fraction (supernatant), and *lane 3* insoluble fraction (pellet). *Arrows* indicate the bands corresponding to HNRDPL protein.

The inherent aggregation propensity of human amyloid proteins results in most of them aggregating into insoluble IBs when they are produced in bacteria [55]. To test if this is the case of HNRPDL, we analyzed the cellular distribution of the recombinant protein after its expression in *E. coli* at 37°C for 20 h. As assessed by SDS-PAGE, a new protein band of ~50 kDa, corresponding to the expected HNRPDL molecular weight (47 kDa), could be detected in induced cells (Figure 2a). The bacteria cells were harvested, lysed and centrifuged and the resulting supernatant and pellet fractions were analyzed by SDS-PAGE. HNRPDL was found essentially in the insoluble fraction suggesting that it likely aggregated into IBs (Figure 2a). The protein remained in the insoluble fraction when protein expression was induced at either 25 or 18°C (data not shown). We further cloned the HNRPDL cDNA downstream of the GST gene in a pETM30 vector and expressed the fusion protein at 20°C for 20 h. A new protein band of ~75 kDa was observed for induced cells, corresponding to the sum of the molecular weights of GST (26 kDa) and HNRPDL (47 kDa) (Figure 2b). Fractionation indicated that despite the theoretical solubility provided by GST, the fusion was located in the insoluble fraction (Figure 2b) a localization that was maintained when protein expression experiments were performed at lower temperatures (data not shown). Because RRM domains are known to be soluble at high concentrations [56] and no aggregation-prone region is detected at the disordered N-terminal segment using predictive algorithms like AGGRESCAN [57] or TANGO [58], it is likely that the predicted amyloidogenicity of the prion-like C-terminal region would account for the propensity of HNRPDL to form intracellular aggregates, either alone or when fused to GST.

### HNRPDL IBs bind to thioflavin-S in living cells

**Figure 3:**
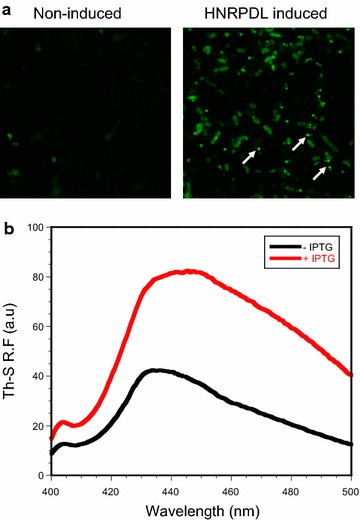
Th-S staining of cells expressing HNRPDL. **a** Fluorescent confocal microscopy images of non-induced *E. coli* cells and expressing HNRPDL IBs stained with Th-S at ×100 magnification. **b** Fluorescence spectra of Th-S in the presence of non-induced (−IPTG) and induced (+IPTG) living cells expressing HNRPDL. *Arrows* indicate the position of IBs.

We have shown recently that thioflavin-S (Th-S) staining of living bacterial cells can be used to detect the presence of intracellular amyloid-like structures as well as to find inhibitors that interfere with amyloid formation [17, 59]. The staining of cells expressing HNRPDL was monitored using confocal microscopy. As it can be observed in Figure 3a, induced cells exhibited a green fluorescent background with strong fluorescent foci located at the cell poles, suggesting that HNRPDL adopts amyloid-like conformations in bacterial IBs. In contrast, non-induced control cells exhibited only residual fluorescence. The presence of intracellular amyloid-like protein conformations in induced cells could also be monitored using fluorescence spectroscopy. As previously described for cells expressing Aβ42 [59], the Th-S fluorescence maximum increases and red-shifts in the presence of living cells expressing HNRPDL, relative to the Th-S fluorescence maximum recorded in the presence of non-induced cells (Figure 3b).

### Purified HNRPDL IBs bind to amyloid dyes

**Figure 4:**
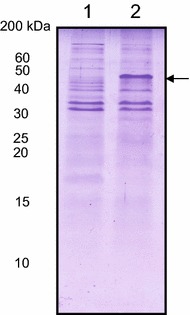
Purification of recombinant HNRPDL IBs. SDS-PAGE analysis of IBs purified from the insoluble fraction of induced cells grown at 37°C containing either an empty plasmid (*lane 1*) or a plasmid encoding for HRNPDL (*lane 2*). The *arrow* indicates the band corresponding to HNRDPL.

We next purified the HNRPDL IBs to characterize biophysically their amyloidogenic properties. Using SDS-PAGE densitometry we calculated that HNRPDL constituted around 30% of all proteins in the purified IBs fraction (Figure 4). To evaluate the specific contribution of HNRPDL in the different assays, relative to that of other proteins present in this fraction, cells bearing the same plasmid without any insert were induced and the IBs fraction purified in the same manner than those containing the HNRPDL cDNA and used as negative control (Figure 4). In addition, the IBs of cells expressing the yeast prion Ure2p and Aβ42 were purified using the same protocol and used as positive controls, since extensive characterization of the bacterial IBs formed by these two proteins have revealed that they posses an amyloid-like nature [16, 27].

**Figure 5:**
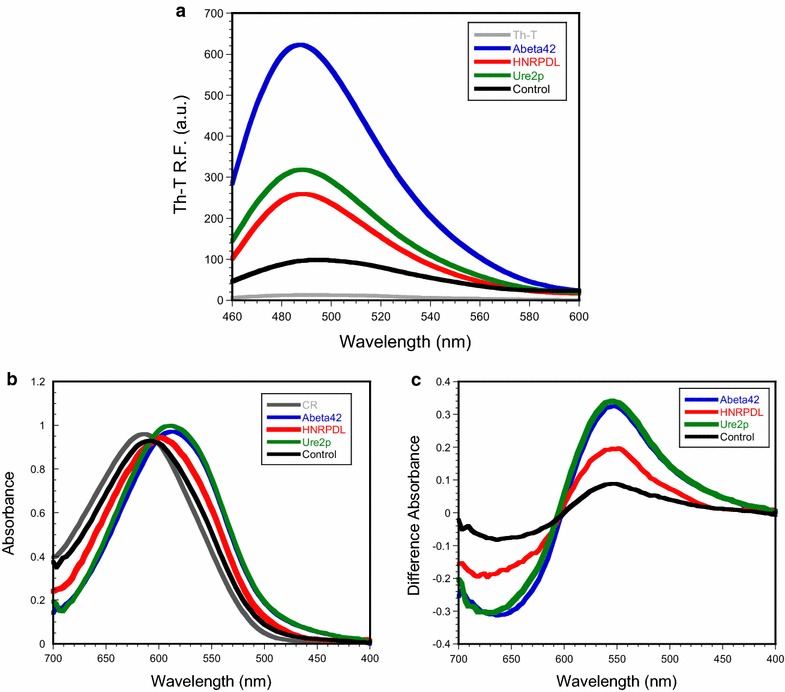
Specific binding of amyloid dyes to HNRPDL IBs. **a** Fluorescence emission spectra of Th-T in the absence or the presence of Aβ42, Ure2p, HNRPDL and control IBs. **b** Congo red (CR) absorbance spectra in the absence or the presence of Aβ42, Ure2p, HNRPDL and control IBs. **c** Difference absorbance spectra of CR in the presence and in the absence of IBs, showing the characteristic amyloid maximum at 540 nm.

Thioflavin-T (Th-T) fluorescence emission is enhanced in the presence of amyloid fibrils [60]. Consistent with their amyloid properties, the same behaviour is observed upon incubation of Th-T with Aβ42 and Ure2p IBs. In the same way, the increase in Th-T fluorescence in the presence of HNRPDL IBs suggests the existence of amyloid conformations in the polypeptides embedded in these aggregates (Figure 5a). Although their impact in Th-T fluorescence is lower than that of Aβ42 IBs, it is quite similar to the one promoted by Ure2p IBs and remarkably different from that observed in the IBs fraction of negative control cells.

The absorbance of the amyloid dye congo red (CR) red-shifts in the presence of amyloid fibrils [61]. The same effect was observed in the presence of Aβ42, Ure2p and HNRPDL IBs, consistent with the presence of amyloid-like structures in these aggregates. The observed red-shift was smaller for HNRPDL than for the other two amyloid proteins, but still significantly different from that promoted by the IBs fraction of negative control cells (Figure 5b). Indeed, quantification of CR bound to IBs (see “Methods”) indicates that HNRPDL binds 2.4 times more dye than control IBs. The difference spectrum between the dye in the absence and presence of purified IBs allows the detection of the characteristic band at 540 nm, corresponding to the amyloid conformation in the three IBs (Figure 5c).

### HNRPDL IBs are enriched in intermolecular β-sheet structure

**Figure 6:**
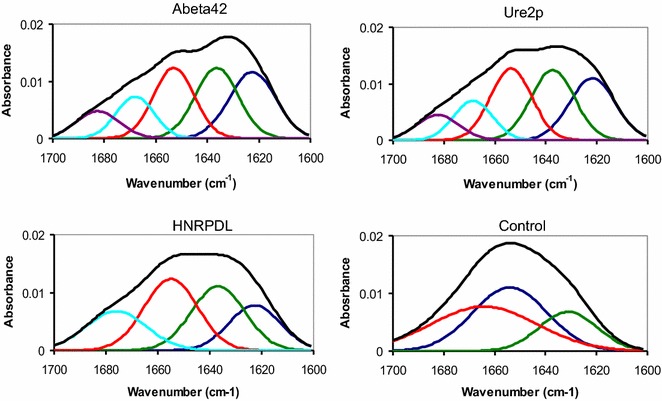
Secondary structure content of HNRPDL IBs. FTIR absorbance in the amide I region of the infrared spectrum (*black*) for Aβ42, Ure2p, HNRPDL and control IBs. Spectral components in the Fourier deconvoluted FTIR spectra are shown. The area and position of the correspondent bands are indicated in Table 2.

From a structural point of view, the formation of amyloid fibrils is always characterized by an enrichement in protein β-sheet content [61]. Attenuated Total Reflectance–Fourier Transform Infrared spectroscopy (ATR-FTIR) is a powerful tool to investigate the secondary structure in protein aggregates [62–65]. We used this technique to analyse the conformational properties of the IBs in the present study (Figure 6; Table 2; Additional file 1: Figure S1). Deconvolution of the absorbance spectra in the amide I region allows to observe a signal at ~1,622 cm^−1^ common to the IBs formed by Aβ42, Ure2p and HNRPDL proteins, which is otherwise absent in negative control samples. This band is usually attributed to the presence of densely packed β-sheet structures, linked by short and strong hydrogen bonds, compatible with the intermolecular contacts in an amyloid fold [62]. Aβ42, Ure2p and HNRPDL IBs also share a band at ~1,636 cm^−1^, which has been typically assigned to intramolecular β-sheet; this band is also present in the negative control, but it contributes less to the total spectral area. In contrast, the negative control IBs exhibits higher contributions at ~1,653 cm^−1^ and ~1,665 cm^−1^, which indicates an enrichment in helical, irregular and turn conformations, relative to Aβ42, Ure2p and HNRPDL IBs. Aβ42 and Ure2p IBs display a band at 1,682 cm^−1^, which is usually assigned to a high frequency β-sheet signal [66]. The lack of this signal, together with the presence of an exclusive band at ~1,676 cm^−1^, attributed to turns [66], suggests that despite sharing an amyloid nature, the fine structural properties of HNRPDL IBs differ from those formed by Aβ42 and Ure2p.

**Table 2:** Contribution of secondary structure components to the absorbance FTIR spectra of Aβ42, Ure2p, HNRPDL and control IBs

Aβ42		Ure2p		HRNPDL		Control		
Band (cm^−1^)	Area (%)	Band (cm^−1^)	Area (%)	Band (cm^−1^)	Area (%)	Band (cm^−1^)	Area (%)	
1,622	26.20	1,621	24.72	1,622	19.40	–	–	Intermolecular β-sheet
1,636	25.88	1,637	26.70	1,636	28.67	1,630	19.32	Intramolecular β-sheet
1,652	24.48	1,653	25.89	1,654	32.00	1,653	40.44	α-helix/random
1,668	13.48	1,668	13.31	–	–	1,667	40.22	Turns
–	–	–	–	1,676	14.92	–	–	β-turns
1,682	9.94	1,682	9.38	–	–	–	–	Antiparalel β-sheet

### HNRPDL IBs posses an inner amyloid core

**Figure 7:**
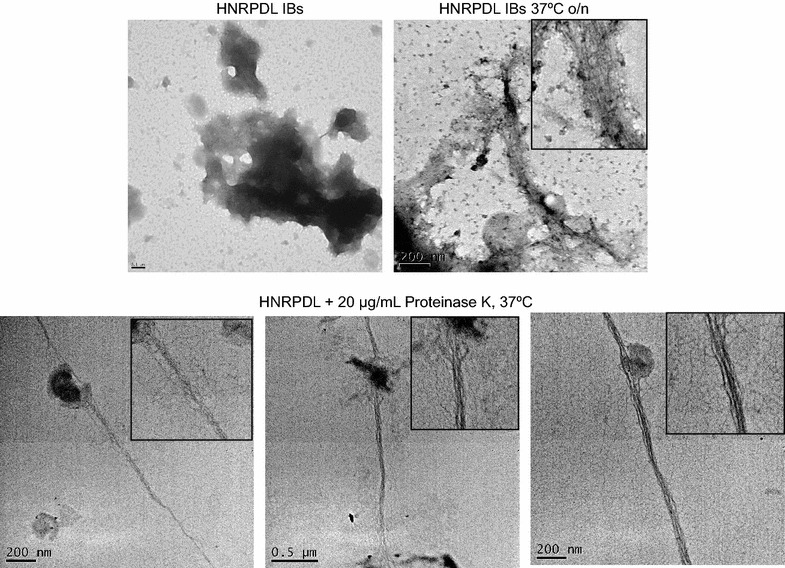
HNRPDL IBs contain amyloid-like fibrils. Negatively stained HNRPDL IBs visualized by TEM. The *upper panel* shows freshly purified HNRPDL IBs (*left*) and IBs incubated overnight at 37°C (*right*). The *bottom panel* displays representative micrographs of PK digested HNRPDL IBs.

We monitored the morphology of HNRPDL IBs using Transmission Electronic Microscopy (TEM). Freshly purified IBs displayed a typical electrodense amorphous appearance (Figure 7). However, upon incubation of purified IBs at 37°C for 12 h, the presence of fibrillar structures becomes already evident (Figure 7). The same behaviour has been reported for the amyloid-like IBs of other proteins and interpreted as the IBs containing densely packed bundles of amyloid fibrils inside cells that become relaxed and exposed upon in vitro incubation [14]. This property can be qualitatively tested using proteinase K (PK), a protease usually used to map the protected core of amyloid fibrils because in spite of being highly active against peptidic bonds it cannot easily attack the highly packed backbones in amyloid β-sheet structures. Accordingly, we have shown that PK digestion allows revealing the existence of a fibrillar core in Aβ peptide IBs [15]. We used the same approach to assess if the presence of a similar fibrillar material might account for the amyloid conformational properties of HNRPDL IBs. Upon PK digestion, the presence of typical long and unbranched amyloid fibrils becomes evident. The fibrils are associated with apparently amorphous material and in some micrographs fibrils emerging from the preformed compact IBs are seen. The elementary fibrils are ~5 nm in diameter and tend to associate laterally into bundles, thus supporting that HNRPDL IBs constitute a bacterial reservoir of amyloid structures, that coexist with less ordered and PK susceptible protein regions, in good agreement with the deduced secondary structure content from FTIR analysis. According to the presence of an amyloid core: (1) HNRPDL IBs are much more resistant towards PK digestion than negative control IBs (Additional file 2: Figure S2) and (2) HNRPDL IBs retain significantly higher Th-T binding in diluted solutions than negative control IBs even upon long time incubation (Additional file 3: Figure S3). These two properties recapitulate that of the amyloid-like IBs formed by Aβ40 and Aβ42 peptides in bacteria [16].

### HNRPDL IBs are toxic to cultured neuronal cells

**Figure 8:**
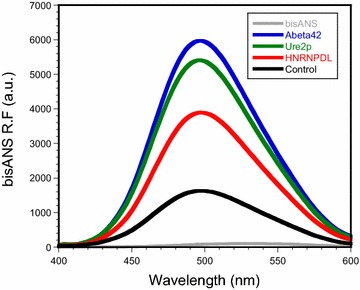
Binding of bis-ANS to HNRPDL IBs. Fluorescence spectra of bis-ANS in the absence and presence of Aβ42, Ure2p, HNRPDL and control IBs.

It has been shown for different and unrelated proteins that the binding to ANS-like dyes correlates with the toxicity of amyloid species, suggesting that the exposure of hydrophobic patches is a critical characteristic of these pathogenic assemblies [67]. We analyzed the binding of bis-ANS to Aβ42, Ure2p and HNRPDL IBs. In the presence of these aggregates, bis-ANS experienced the expected blue-shift and a strong increase in fluorescence maximum. The strongest spectral changes were promoted by the Aβ42, and the prion Ure2p IBs. However, HNRPDL IBs induced a significantly higher increase in bis-ANS fluorescence than negative control IBs (Figure 8).

**Figure 9:**
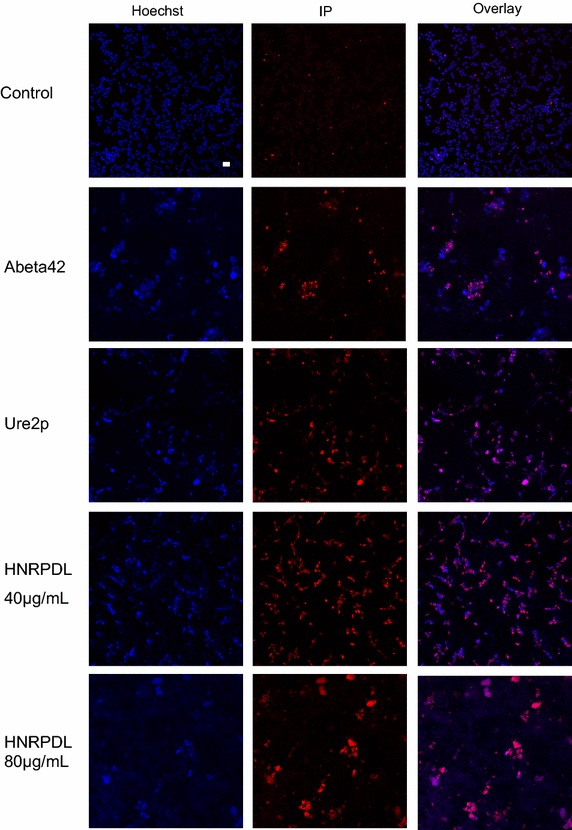
Toxicity of HNRPDL IBs as visualized by confocal microscopy. Representative confocal fluorescence microscopy images of SH-SY5Y cells stained with propidium iodide (IP) or Hoechst after incubation with Aβ42, Ure2p, HNRPDL and control IBs for 24 h at 37°C. The *bar* corresponds to 15 µM.

The aggregates formed by different human prion-like proteins have been shown to exert neurotoxicity [68]; therefore we tested if, in agreement with their bis-ANS binding ability, purified HNRPDL IBs could be toxic for cultured neuroblastoma SH-SY5Y cells. The combination of Hoechst and propidium iodide (PI) staining allows to asses cell viability by fluorescence microscopy, as viable cells are permeable to Hoechst and PI only enters cells with disintegrated membranes thus corresponding to dead cells. Cell morphology can be monitored as well to discriminate toxic and non-toxic aggregates in this assay. In samples treated with negative control IBs cell were attached to the culture plate at a confluent stage with only a reduced number of cells becoming stained with PI, indicating that they display low or no toxicity (Figure 9). In contrast, the IBs formed by Aβ42 and Ure2p proteins were inherently toxic to neuronal cells as both induce positive PI staining in most cell nuclei (Figure 9). In the same manner, HNRPDL IBs turned to be highly neurotoxic, with a large majority of cells being stained by PI (Figure 9). Moreover, this effect was dose dependent, since cells incubated with 40 µg/mL of HNRPDL IBs kept attached, homogeneously distributed and displayed normal morphology, whereas cells treated with 80 µg/mL HNRPDL IBs lost completely their morphology becoming detached and agglutinated (Figure 9).

## Discussion

The number of human proteins involved in neurodegenerative disorders is rapidly expanding, suggesting that there are likely numerous disease-associated proteins yet to be identified. Many of these disorders involve the formation of self-templating aggregates [69]. However, since most protein aggregates are not infectious, prion-based disorders have been always considered different from the rest of aggregation caused diseases. Nevertheless, increasing evidence indicates that the proteins involved in many neurodegenerative disorders, including Alzheimer’s and Parkinson’s, display a prion-like behaviour, exhibiting a cell-to-cell propagation [70]. In addition, different human proteins containing intrinsically disordered domains with an amino acid composition resembling those of the prion forming domains (PFDs) in yeast prions are being found connected to degenerative disorders [71]. Many of these disorder-linked PrLD-containing proteins are RNA-binding proteins typically containing one or more RRM domains [37]. TDP-43 was the first identified protein of this class. It was initially found to be a major constituent of the protein aggregates in the spinal cord motor neurons, in the hippocampus and neocortex of ALS or FTLD patients, but it is also present in an aggregated form in other neurodegenerative disorders [39]. A majority of the mutations linked to ALS or FTLD map into the PrLD, implicating thus this domain in the disease [71]. HNRPDL is a less studied RNA-binding protein, which shares domain organization with TDP-43 (Figure 1), despite its precise three-dimensional structure is unknown. Interestingly, it has been shown that two mutations occurring in the PrLD of this protein, D378N and D378H, lead to limb-girdle muscular dystrophy 1G [47]. According to PrionScan, PLAAC and PAPA prion predictors [52–54] these two mutations increase the prion propensity of the domain (Table 3).

**Table 3:** Predicted prion propensity of wild type HNRPDL and mutants involved in limb-girdle muscular dystrophy 1G

	PrionScan [54]	PAPA [52]	PLAAC [53]
HNRPDL	42.904	0.12	30.301
D378H	44.280	0.14	31.395
D378 N	46.922	0.15	33.013

The structure of TDP-43 inclusions in ALS and FTLD patients is still unclear and whether these deposits have an amyloid nature or not is matter of debate. Due to the difficulty of purifying TDP-43 for the in vitro characterization of its aggregation process and because the intracellular aggregation of human amyloid proteins in bacteria has been shown to result into amyloid-like IBs, Chiti and co-workers characterized the nature of the IBs formed by TDP-43 in *E. coli* to approximate the conformational properties of its inclusions in ALS and FTLD [45]. They found out that TDP-43 aggregates present in *E. coli* IBs did not possess any of the hallmarks of amyloid fibrils, allowing them to be classified as amorphous. However, they were shown to be toxic for cultured neuronal cells. This raises the question of whether this conformation is a generic property of the aggregates formed by human PrLD-containing proteins and whether it is indeed the lack of an ordered structure in the aggregates the underlying cause of their toxicity in pathological states. The analysis of the conformational aggregates formed by HNRPDL in bacteria indicate that this is not the case, since these aggregates bind to amyloid dyes, are enriched in intermolecular β-sheet conformation and contain inner fibril-like structure; still they are neurotoxic. The amorphous nature of the aggregates formed by TDP-43 contrasts with those formed by the yeast PFDs to which its PrLD resembles, since these latter display clear amyloid properties, both in vitro [72] and when expressed recombinantly in bacteria [27]. We have shown that the presence of a short amyloidogenic stretch in PrLDs, as predicted with our algorithm pWALTZ, determines to a large extent its amyloid potential [34]. Q/N enriched yeast putative prion domains with pWALTZ scores higher than 73.55 all formed amyloid assemblies, whereas those falling below this threshold display lower amyloid propensity. Interestingly, the PrLDs of HNRPLD and TDP-43 display pWALTZ values above and below this threshold, respectively, which might account, at least in part, for their different intracellular amyloid propensity.

Aggregation constraints the evolution of proteins and accordingly nature have evolved different strategies to minimize protein aggregation in sequences and structures [73]. In this context, the inherent aggregation of human proteins containing PrLDs and their link to disease, strongly suggest that these domain are conserved because they serve functional purposes. Increasing evidence indicates that in RNA-binding proteins, these disordered domains work in the reversible recruitment of the protein into RNA-P bodies or stress granules under cellular stress [38, 74]. The amyloidogenic properties of HNRPLD constitute yet another example illustrating how the determinants for the establishment of functional interactions and those accounting for the formation of toxic amyloid assemblies overlap significantly [75, 76], suggesting that in PrLDs-containing proteins the formation of functional macromolecular complexes and the aggregation of their individual subunits might compete in the cell. This will explain, why point mutations in these domains or environmental changes, such as prolonged stress, enhance recruitment into stress granules [43, 77], disrupting the reversibility of the assembly and finally leading to the accumulation of aggregates, triggering the onset of the disease. The present work illustrates the potency of microbial cell factories to model amyloid conformational conversion.

## Methods

### Protein expression and purification

Human HNRPDL cDNA was cloned into a pET28a(+) vector (Novagen, INC., Madison, WI, USA). The plasmids encoding for Aβ42 and Ure2p proteins were as previously described [16, 27, 78]. The plasmids were transformed into *E. coli* BL21(DE3) cells. Cells were grown aerobically in liquid Luria–Bertani (LB) medium containing appropriate antibiotics in a rotary shaker at 37°C and 250 rpm. Overnight cultures were diluted 100-fold in LB and allowed to grow to an OD_600_ of 0.6. At the indicated OD_600_, protein expression was induced with 1 mM isopropyl β-d-1-thiogalactopyranoside (IPTG) and in the case of Aβ42 and Ure2p the culture was continued at 37°C for 4 h as previously described [16, 78]. HNRPDL cells were cultured at 37°C 25°C or 18°C for 20 h upon induction. To express HNRPDL-GST, the human HNRPDL sequence was cloned into a pETM-30 vector in order to produce a N-terminal fusion protein with a His tag followed by GST with a TEV protease cleavage site; the resulting construct was transformed into *E. coli* BL21(DE3) cells and grown as described above, inducing protein expression for 20 h at 20°C or 16°C. As a negative control, *E. coli* BL21(DE3) cells were transformed with an empty pET28a(+) vector, grown and induced in the same conditions than cells containing the HNRPDL encoding plasmid.

### Inclusion bodies purification

Intracellular IBs were purified as previously described [15]. Briefly, cell pellets from 5 mL induced cultures were resuspended in 140 μL of lysis buffer (10 mM Tris–HCl, pH 8.0, 1 mM EDTA, 150 mM NaCl), containing 0.8 μL protease inhibitor PMSF (17.4 mg/mL) and 3 μL lysozyme (10 mg/mL). The suspension was incubated for 30 min at 37°C under gentle agitation. Then cells were incubated with 1% (v/v) NP-40 for 50 min under mild agitation at 4°C. To remove nucleic acids, 3 μL of DNase I from a 1 mg/mL stock, 3 μL of 1 mg/mL of RNase and 3 μL of 1 M MgSO_4_ were added and the resulting mixtures were further incubated at 37°C for 30 min. IBs were collected by centrifugation at 12,000×*g* for 15 min at 4°C. Finally, IBs were washed with lysis buffer containing 0.5% Triton X-100 three times, twice with lysis buffer and finally stored at -80°C until analysis. The purified IBs fraction was resolved on a 15% SDS–PAGE gel stained with Coomassie brilliant blue.

### Thioflavin-S binding in living cells

Detection of cell-permeable thioflavin-S (Th-S) binding was performed in non-induced and induced living cells expressing HNRPDL protein. Bacterial cells were washed with PBS and diluted to an OD_600nm_ of 0.1. Cells were incubated for 1 h in the presence of 125 µM Th-S diluted in PBS and washed twice with PBS. Fluorescence emission spectra were recorded in a range of 400–500 nm using an excitation wavelength of 375 nm. Apertures of 5 nm were fixed in both excitation and emission slits. The analysis of fluorescence microscope images allowed the detection of accumulated amyloid deposits inside bacterial cells. Cells were placed on top of a microscope slide and covered with a cover slip. Photographs were acquired using a 488-nm argon laser and emission collected in a 515–540 nm range.

### Thioflavin-T binding

Thioflavin-T (Th-T) binding was analyzed for IBs purified from cells expressing Aβ42, Ure2p or HNRPDL and from control cells, resuspended in PBS at pH 7.0 and OD_350nm_ of 0.1 in the presence of 25 μM Th-T. Fluorescence emission spectra were recorded from 460 to 600 nm with an excitation wavelength of 440 nm, using a slit width of 5 nm for excitation and emission in a Jasco FP-8200 spectrophotometer (Jasco corporation, Japan). Each trace represents the average of 3 accumulated spectra.

Th-T fluorescence kinetics for HNRPDL and negative control IBs were analyzed from diluted IBs at a final OD_350nm_ of 0.05 in PBS at pH 7. Samples were incubated for 400 min under agitation (800 rpm) at 25°C, in the presence of 25 μM Th-T. The kinetic traces were measured exciting at 440 nm and emission was recorded at 475 nm, slit width of 5 nm were used for excitation and emission in a Jasco FP8200 spectrophotometer (Jasco corporation, Japan).

### Congo red binding

Congo red (CR) interaction with IBs purified from cells expressing Aβ42, Ure2p or HNRPDL and from control cells was tested using a Cary-400 UV/Vis spectrophotometer. IBs samples were diluted to a final OD_350nm_ of 0.1 in PBS at pH 7.0 and 20 μM of CR was added. After 5 min of equilibration, the absorbance spectra were recorded from 400 to 700 nm. The differential CR spectra in the presence and absence of protein were calculated to detect the typical amyloid band at ~540 nm. CR binding was quantified by the equation: CR Bound = Abs_540nm_/25,295 − Abs_477nm_/46,306.

### Bis-ANS binding

Binding of 4,4’-bis[1-anilinonaphthalene 8-sulfonate] (bis-ANS) to purified Aβ42, Ure2p, HNRPDL IBs and the negative control extract was evaluated by registering bis-ANS fluorescence between 400 and 600 nm after excitation at 370 nm in a Jasco FP-8200 spectrophotometer (Jasco corporation, Japan), with excitation and emission slit widths of 5 nm. 25 μM of bis-ANS was added to IBs at a final OD_350_ of 0.1 in PBS. Spectra were registered at 25°C as the accumulation of three consecutive scans, after equilibration of the sample for 5 min.

### ATR-FTIR spectroscopy

ATR FTIR spectroscopy analyses of purified Aβ42, Ure2p, HNRPDL and control IBs were performed with a Bruker Tensor 27 FTIR Spectrometer (Bruker Optics Inc.) with a Golden Gate MKII ATR accessory. Spectrum acquisitions consisted of 16 independent scans, measured at a resolution of 2 cm^−1^ within the 1,800–1,500 cm^−1^ range. Spectra were acquired, background subtracted, baseline corrected and normalized using the OPUS MIR Tensor 27 software. Second derivatives of the spectra were used to determine the frequencies at which the different spectral components were located. All FTIR spectra were fitted to overlapping Gaussian curves using PeakFit package software (Systat Software) and the maximum and the area of each Gaussian were calculated.

### Limited proteinase K digestion

HNRPDL and negative control IBs were resuspended at a final OD_350_ of 1 in PBS buffer at pH 7.0. Digestion was initiated by adding proteinase K (PK) at a final concentration of 20 μg/mL and the reaction was carried out for 30 min at 37°C under agitation (500 rpm). PK proteolysis was monitored at 350 nm using a Cary-400 UV/Vis spectrophotometer.

### Transmission electron microscopy (TEM)

Purified HNRPDL IBs (100 µg/mL) were digested with 20 μg/mL proteinase K (PK) and incubated at 37°C at different digestion times. Proteolytic mixtures were centrifuged and pellets were resuspended in water. Then 10 μL of purified and PK digested HNRPDL IBs solutions were placed on carbon-coated copper grids and allowed to stand for 5 min. For negative staining, grids were washed with distilled water and stained with 2% (w/v) uranyl acetate for 1 min. The samples were imaged using a JEM-1400 transmission electron microscope operating at an accelerating voltage of 120 kV.

### Cell viability assay

Human SH-SY5Y cells were cultured in F-12 medium supplemented with 10% FBS on glass slides at 70% confluence and maintained at 37°C in a 5% CO_2_ atmosphere. Cell cultures were incubated in the absence (control) and the presence of Aβ42, Ure2p and HNRPDL IBs resuspended in sterile PBS for 24 h. Cells were counterstained with 0.5 μg/mL Hoechst and 10 μg/mL PI (Molecular Probes) for 15 min at 37°C and washed twice with PBS buffer. Cell morphology and viability were analyzed by confocal fluorescence microscopy (Olympus Fluoview 1000) with an UPlansApo 10x objective using an orange diode (588–715 nm emission collected) and a UV laser (excited at 350 nm and collected at 405 nm).

## Additional files

Additional file 1:
**Figure S1.** FTIR absorbance (up) and second derivative of the FTIR absorbance in the amide I region of the infrared spectrum for Aβ42, Ure2p, HNRPDL and control IBs Additional file 2:
**Figure S2.** Kinetics of HNRPDL and control IBs digestion by 20 µg/mL PK at 37 °C followed by the decrease in turbidity at OD_350nm_. Note how the turbidity of control IBs decreases suddenly upon PK addition whereas in HNRPDL IBs the signal is lost more progressively, indicating higher resistance to proteolysis Additional file 3:
**Figure S3.** Th-T fluorescence kinetics for HNRPDL and control IBs. In both cases, the initial stock solutions were diluted to yield a final OD_350nm_ of 0.05 in a 25 µM Th-T containing solution at 25 °C with continuous agitation. Control IBs bind little Th-T already at the beginning of the experiment and the signal decays linearly with time. In contrast the Th-T fluorescence of HNRPDL IBs shows a biexponential behaviour with measurable fluorescence still after 6 h, indicative of the presence of a persistent amyloid structure

## Abbreviations

ATR-FTIR: attenuated total reflectance–fourier transform infrared spectroscopy
ALS: amyotrophic lateral sclerosis
bis-ANS: 4,4’-bis[1-anilinonaphthalene 8-sulfonate]
CR: congo red
EWSR1: EWS RNA-binding protein 1
FTLD: frontotemporal lobar degeneration
FUS: fused in sarcoma
GST: glutathione S-transferase
hnRNP: heterogenous ribonucleoprotein
HNRPDL: heterogenous nuclear ribonucleoprotein D-like
IBs: inclusion bodies
IPTG: isopropyl β-d-1-thiogalactopyranoside
PAPA: prion aggregation prediction algorithm
PBS: phosphate buffer saline
PI: propidium iodide
PK: proteinase K
PLAAC: prion-like amino acid composition
PrLDs: prion like domains
RRM: RNA recognition motif
RONN: regional order neural network software
TAF15: TATA-binding protein-associated factor 2 N
TDP-43: TAR DNA-binding protein 43
TEM: transmission electronic microscopy
Th-S: thioflavin-S
Th-T: thioflavin-T
