# Supplementary figures and images for: The prion-like RNA-processing protein HNRPDL forms inherently toxic amyloid-like inclusion bodies in bacteria
Source: Microb Cell Fact. 2015 Jul 11;14:102. doi: 10.1186/s12934-015-0284-7 (PMC4498515; doi:10.1186/s12934-015-0284-7)

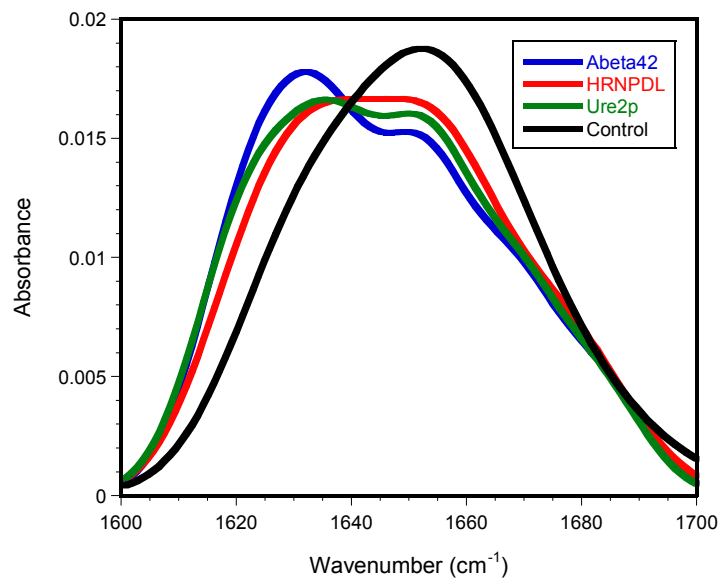

**Abeta42**

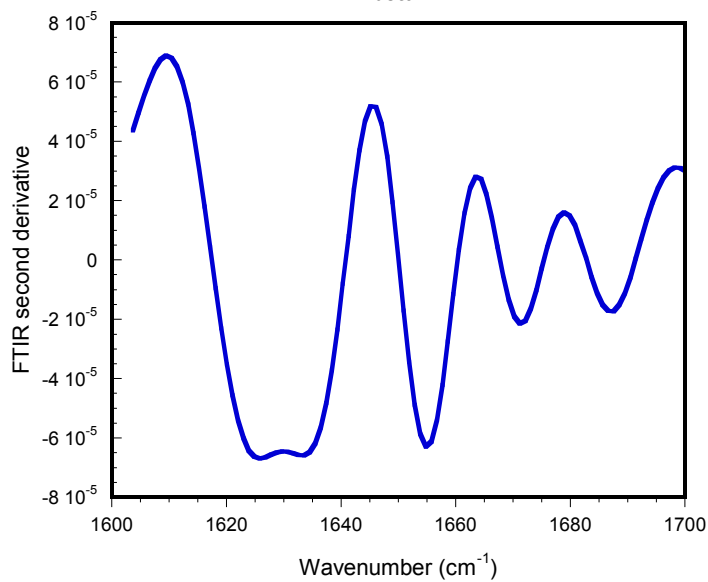

**Ure2p**

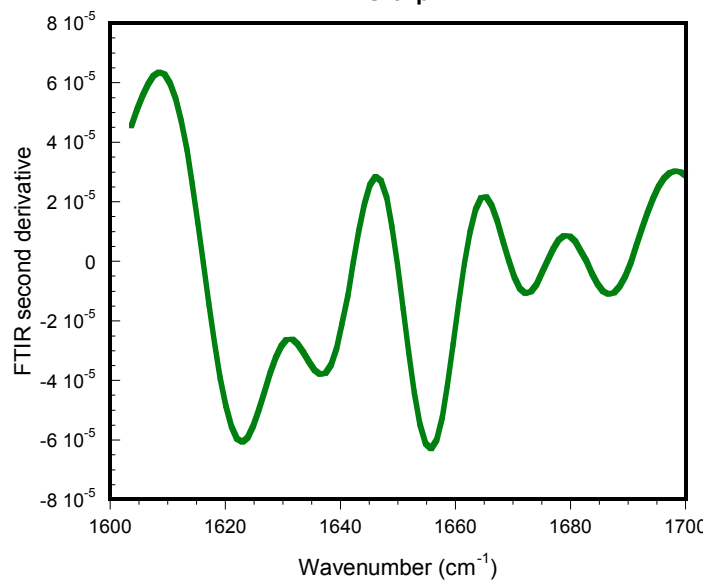

**HRNPDL**

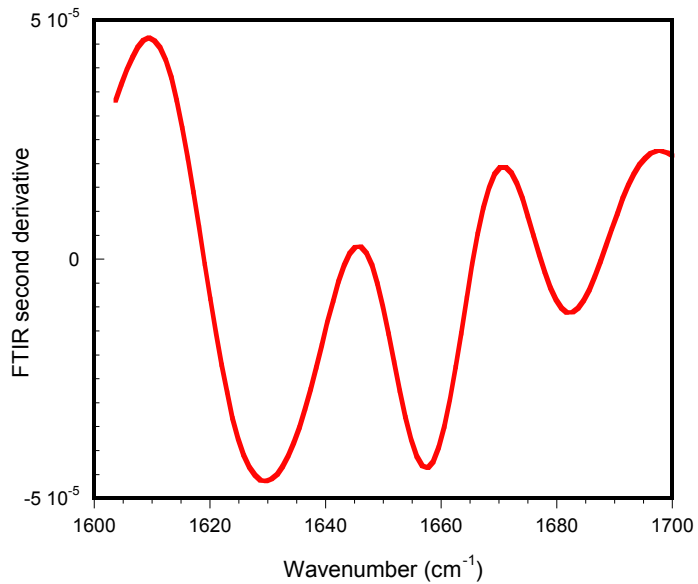

**Control**

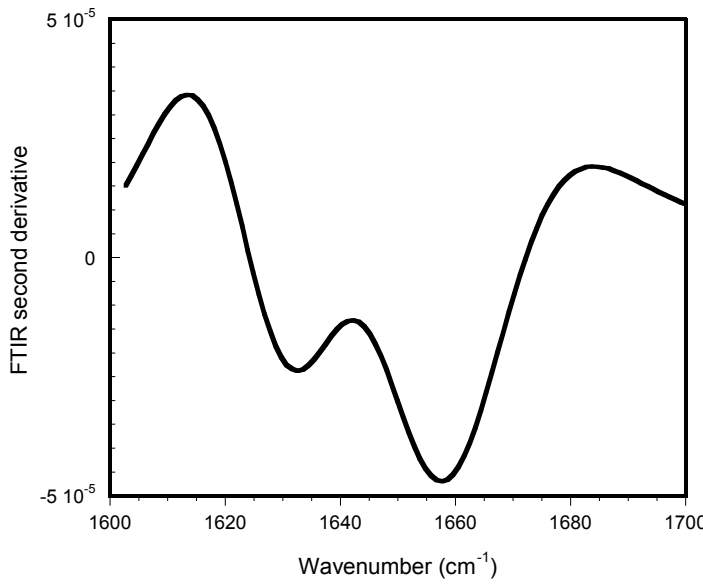

Supplement: Additional file 1: — Figure S1. FTIR absorbance (up) and second derivative of the FTIR absorbance in the amide I region of the infrared spectrum for Aβ42, Ure2p, HNRPDL and control IBs [file 12934_2015_284_MOESM1_ESM.pdf]

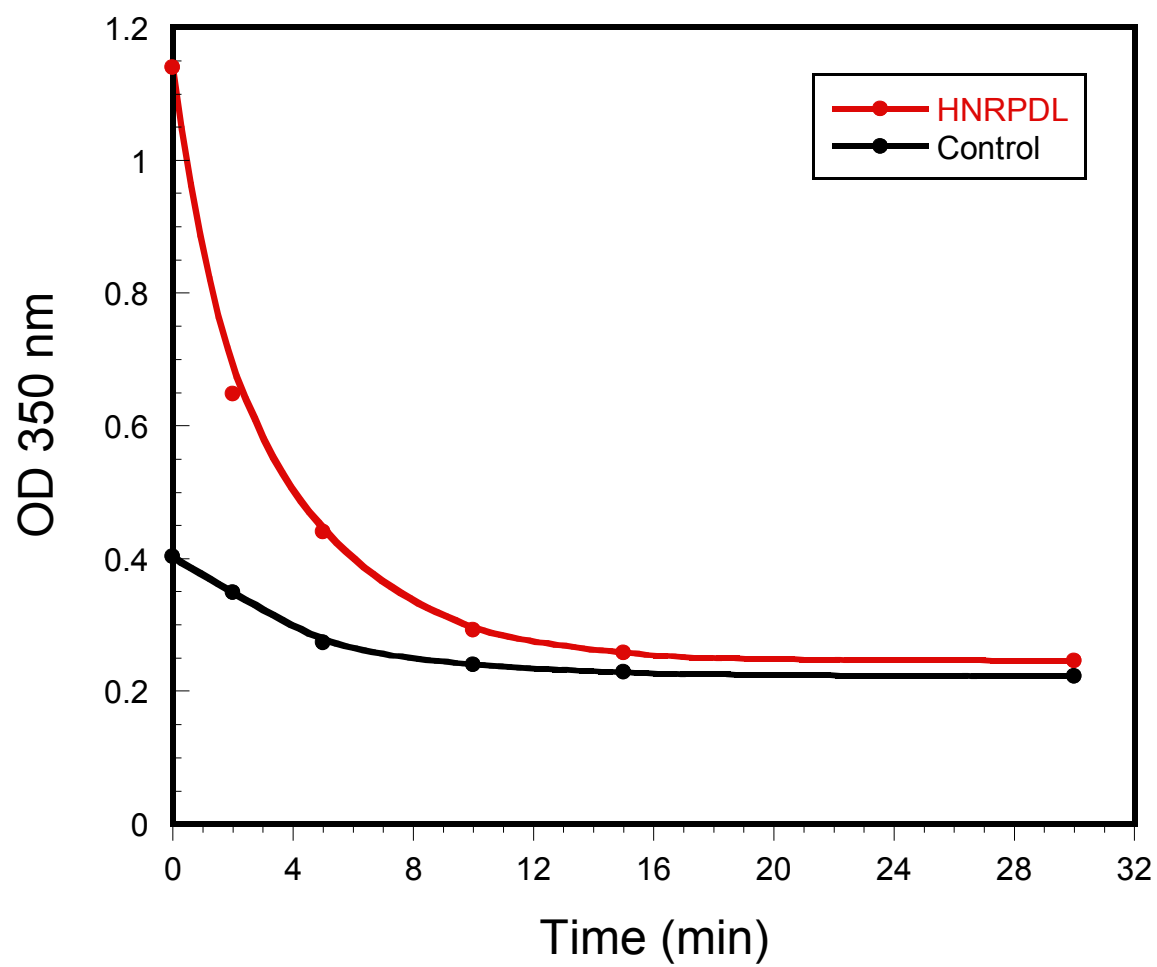

Supplement: Additional file 2: — Figure S2. Kinetics of HNRPDL and control IBs digestion by 20 µg/mL PK at 37 °C followed by the decrease in turbidity at OD350nm. Note how the turbidity of control IBs decreases suddenly upon PK addition whereas in HNRPDL IBs the signal is lost more progressively, indicating higher resistance to proteolysis [file 12934_2015_284_MOESM2_ESM.pdf]

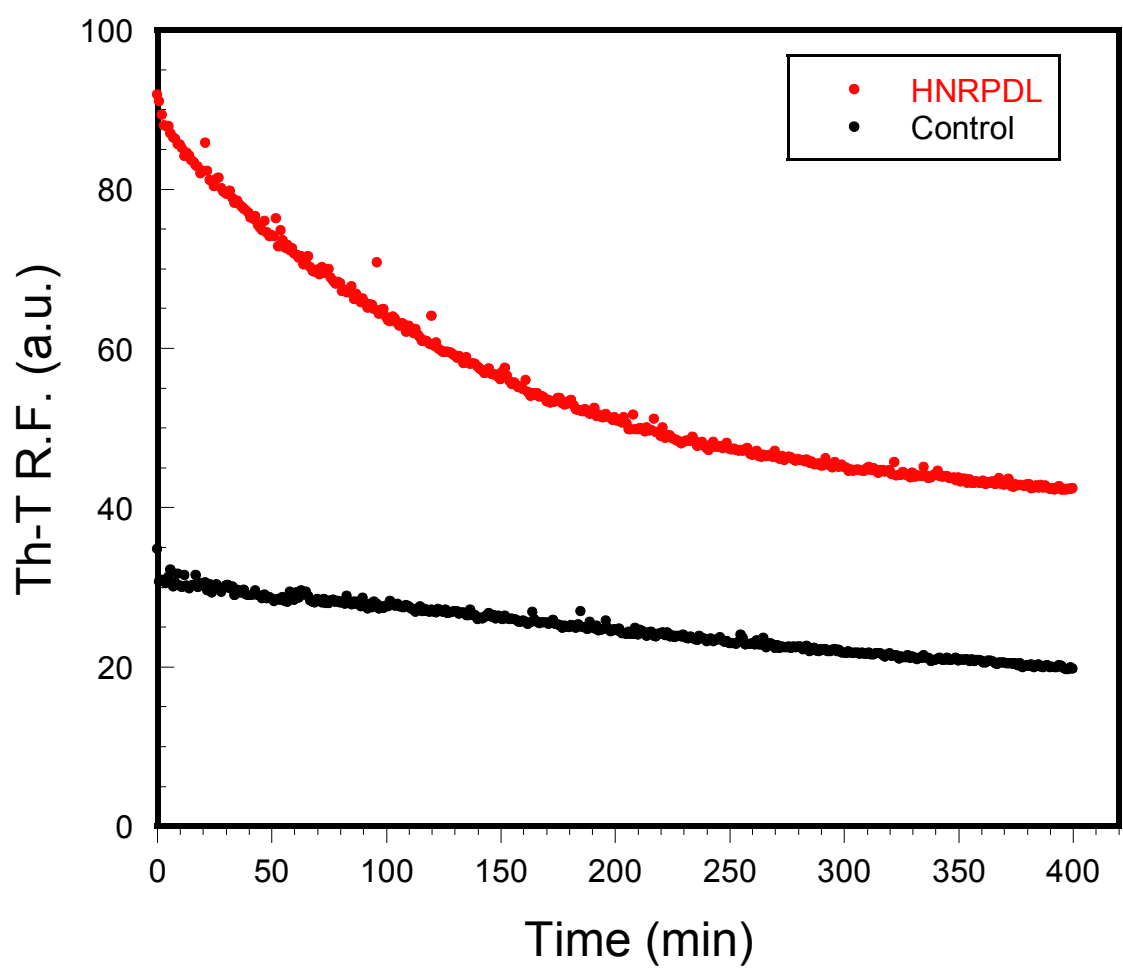

Supplement: Additional file 3: — Figure S3. Th-T fluorescence kinetics for HNRPDL and control IBs. In both cases, the initial stock solutions were diluted to yield a final OD350nm of 0.05 in a 25 µM Th-T containing solution at 25 °C with continuous agitation. Control IBs bind little Th-T already at the beginning of the experiment and the signal decays linearly with time. In contrast the Th-T fluorescence of HNRPDL IBs shows a biexponential behaviour with measurable fluorescence still after 6 h, indicative of the presence of a persistent amyloid structure [file 12934_2015_284_MOESM3_ESM.pdf]
